# Supplementary material for: The interactome of intact mitochondria by cross-linking mass spectrometry provides evidence for coexisting respiratory supercomplexes
Source: Mol Cell Proteomics. 2017 Dec 8;17(2):216–32. doi: 10.1074/mcp.RA117.000470 (PMC5795388; doi:10.1074/mcp.RA117.000470)
Supplement: Supplemental Data [file supp_RA117.000470_133922_0_supp_23978_fzffwf.zip › spectra_annotation/mito_spectra_annotation/492-1-9-1-12-1.pdf]

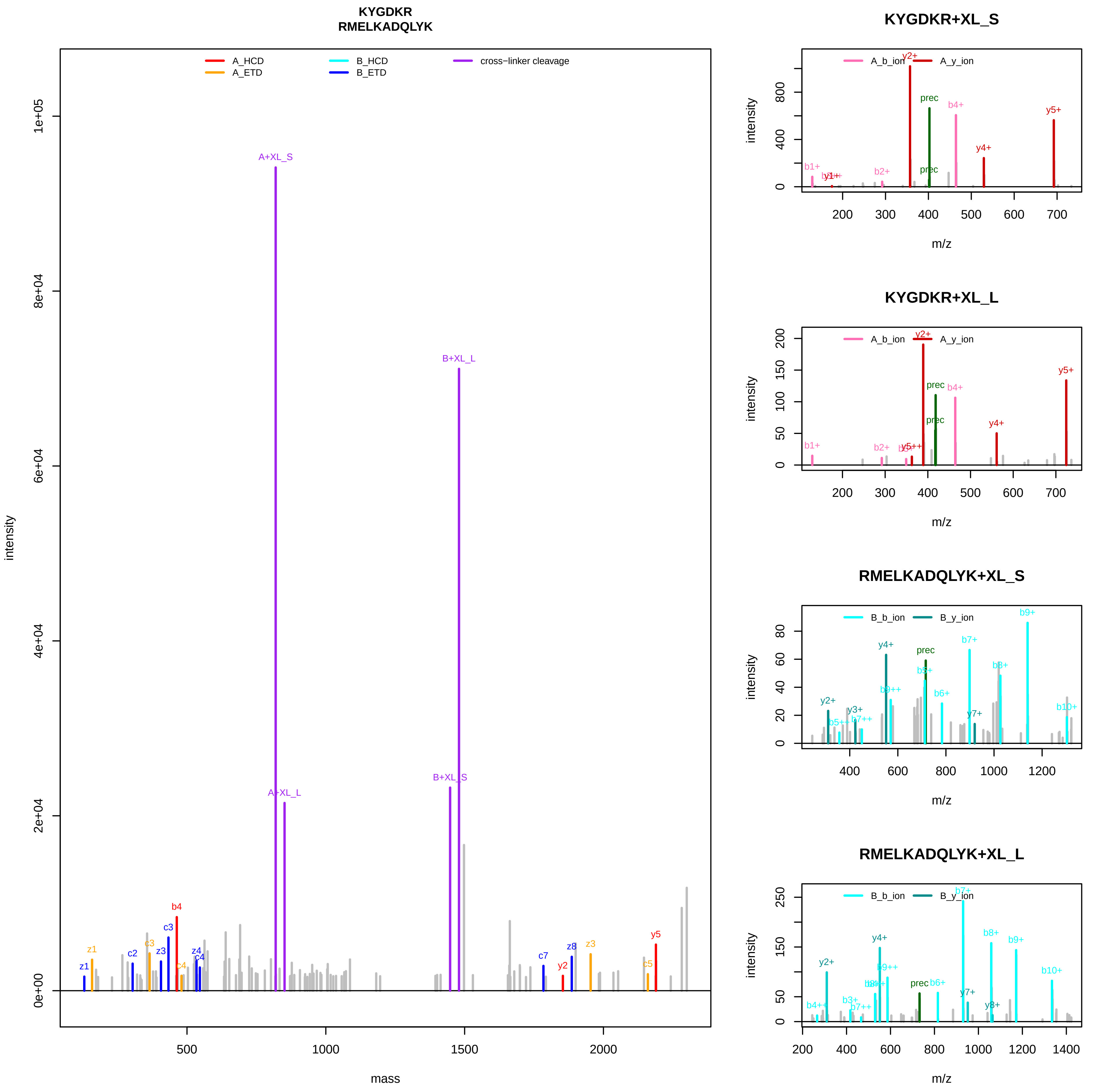
KYGDKR
RMELKADQLYK
A_HCD
A_ETD
B_HCD
B_ETD
cross−linker cleavage
A+XL_S
B+XL_L
A+XL_L
B+XL_S
intensity
mass
0e+00
2e+04
4e+04
6e+04
8e+04
1e+05
500
1000
1500
2000
z1
c2
z3
c3
b4
c4
z4
c7
z8
y2
z3
c5
y5
KYGDKR+XL_S
A_b_ion
A_y_ion
y2+
prec
b4+
y5+
y4+
b1+
b2+
m/z
KYGDKR+XL_L
y5++
RMELKADQLYK+XL_S
B_b_ion
B_y_ion
b9+
y4+
b7+
b8+
b6+
y2+
y3+
b10+
y7+
RMELKADQLYK+XL_L
b7+
y4+
b8+
b9+
b10+
prec
b6+
y7+
y8+
b3+
b4++
m/z
